# Development of an LC-MS method for the semiquantitative determination of polyamide 6 contaminations in polyolefin recyclates

**DOI:** 10.1007/s00216-020-03071-z

**Published:** 2020-11-26

**Authors:** Andrea Schweighuber, Markus Gall, Jörg Fischer, Yi Liu, Hermann Braun, Wolfgang Buchberger

**Affiliations:** 1 Institute of Analytical Chemistry, Johannes Kepler University, 4040 Linz, Austria; 2 Institute of Polymeric Materials and Testing, Johannes Kepler University, 4040 Linz, Austria; 3 CES – Circular Economy Solutions – Innovation & Technology, Borealis Polyolefine GmbH, 4021 Linz, Austria

**Keywords:** Polyolefins, Recyclates, Polyamide contamination, Liquid chromatography, Mass spectrometry

## Abstract

Recycling will be of increasing importance in the future, especially for plastic packaging waste mainly consisting of polyolefins. One major problem of recyclates comprises impurities which can have a significant negative impact on future product properties. Polyamide 6 can be found widely as contaminant in recycled polyolefins, leading to a need of quantification methods thereof. In this paper, a method development for the quantitative analysis of polyamide 6 is presented based on analysing ε-caprolactam and related cyclic oligomers as marker compounds in model recyclates of high- and low-density polyethylene and polypropylene compounded with low amounts of polyamide 6. For the method development and tentative identification of the different cyclic compounds, a HPLC-QTOF-MS was used and it was possible to detect six different compounds, ε-caprolactam and the corresponding cyclic di- to hexamer. The quantification was performed with a HPLC-QQQ-MS, equipped with a HILIC column, after sample preparation via microwave-assisted extraction. It could be shown that a good linearity from 0.2 up to 5 wt% polyamide 6 in the different polyolefins can be achieved. The cyclic trimer and tetramer show a low limit of quantification and are therefore well-suited for the quantification, whereas the other cyclic compounds can be then used as qualifiers to avoid false positives. To guarantee the applicability of the method, six real recyclate materials were analysed, whereby in three of them low amounts of polyamide 6 could be detected.

**Supplementary Information:**

The online version contains supplementary material available at 10.1007/s00216-020-03071-z.

**Figure:**
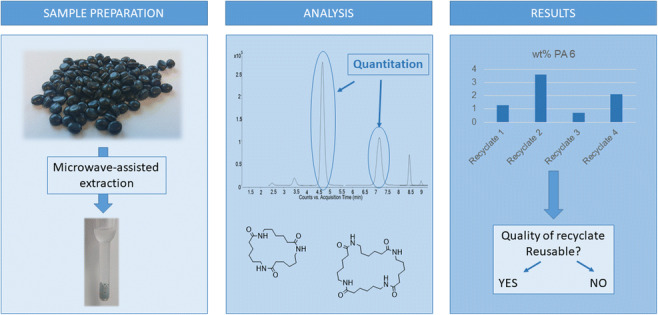
Graphical abstract

## Introduction

In the recent decades, the importance of plastic packaging waste recycling has grown rapidly since the amount of generated waste increased by 19% from 2006 to 2018 in the European Union (EU), leading to a total of 17.8 million tons in 2018. Thereof, 42% were recycled, 39.5% were used for energy recovery and 18.5% were disposed at landfills [1]. To rise the share of recycling, the EU legislated the Directive (EU) 2018/852, regulating minimum plastic recycling quotas of 50% by 2025 and of 55% by 2030 respectively [2]. Mechanical recycling is the main recycling technique, including the steps separation, washing, melting and processing. A crucial part herein belongs to the sorting in order to guarantee pure recyclates without contaminations of other polymers [3–6].

Plastic packaging waste mainly consists of three polymer types, polypropylene (PP), high- and low-density polyethylene (HDPE, LDPE), yet they are frequently processed with other polymers (for example multilayer films), leading to major challenges in separation [1, 3, 7, 8]. According to Faraca et al. [3], 11% of hard plastic and 8% of plastic film waste contained products consisting of more than one polymer type. In 2016, 15.3% of simple multilayer products and 4.3% of thermoformed packaging consisted of polyolefin/polyamide blends [7]. Polyamide 6 (PA 6) possesses several advantages, like heat resistance, high strength and good thermo-formability, which are favourable aspects for packaging applications. This leads to PA 6 being one of the major contaminants in PE and PP recyclates [7–10].

Polymer contaminations present in the recyclates influence mechanical properties and may even lead to phase separations and product failures, due to structure and polarity differences [3–5, 11–14]. Therefore, the quality control with regard to the purity of the recyclates is of utmost importance for the further usage. The main analysis methods comprise melt flow index (MFI), Fourier transform infrared spectroscopy (FTIR), differential scanning calorimetry (DSC) and thermogravimetric analysis (TGA) [11, 14, 15]. Nevertheless, these techniques encounter limitations concerning qualitative and quantitative information of polymer contaminations, for example due to the fact that changes of MFI or DSC/TGA curves can have multiple reasons [11, 14].

It is well known that PA 6 contains small amounts of cyclic oligomers that had been within the focus of studies dealing with their migration from PA food contact materials into food simulants [16–18]. In addition, methods have been developed to measure the content of these oligomers in PA 6 raw material and PA food contact materials [16, 17, 19, 20]. Methods used so far for those purposes were mainly based on liquid chromatography hyphenated with mass spectrometry.

Regarding polyolefin recyclates contaminated by small amounts of PA 6, it may be a promising idea to analyse PA 6 oligomers as markers for the PA contamination. However, extraction techniques so far reported for PA materials are less suited for polyolefin materials. In addition, quantitation limits would have to be much lower than for pure PA materials in order to allow a quantitation of the oligomers from the small amounts of PA 6 contaminations in the polyolefin recyclates. Therefore, the present work aims at the optimization of extraction methods and LC-MS procedures to allow a routine application to recycled polyolefin materials. The analytical approach included microwave-assisted extraction followed by LC-MS using a HILIC column.

## Materials and methods

### Chemicals

Toluene, methanol and acetonitrile were of analytical reagent grade and purchased from VWR International GmbH (Darmstadt, Germany). Ammonium formate (97%) was purchased from Sigma-Aldrich Handels GmbH (Vienna, Austria) and formic acid (> 99%) from VWR International GmbH (Darmstadt, Germany). Water (18 MΩ cm) was obtained from a Millipore purification system (Molsheim, France). The used polymers, HDPE, LDPE, PP and PA 6, were commercially available from local manufacturers. Various products made from recycled polyolefins were bought from local hardware stores.

### Instrumentation

Method development and identification of the analytes were performed on an Agilent 1260 HPLC coupled to an Agilent model 6510 quadrupole time-of-flight MS (QTOF-MS) equipped with an electrospray ionization source (Agilent Technologies, Santa Clara, CA). The samples were measured in positive mode. Hereby, the following parameters were used: 300 °C drying gas temperature, 10 L min^−1^ drying gas flow, 40 psig nebulizer gas pressure and 4000 V capillary voltage.

The quantitative measurements were performed on an Agilent 1260 HPLC coupled to an Agilent model 6460 triple quadrupole MS (QQQ-MS) equipped with an electrospray ionization source (Agilent Technologies, Santa Clara, CA) in MRM mode. The samples were measured in positive mode. For signal enhancement, a source optimization was applied, resulting in the following parameters: 215 °C gas temperature, 10 L min^−1^ gas flow, 40 psig nebulizer, 350 °C sheath gas temperature, 11 L min^−1^ sheath gas flow, 4000 V capillary voltage and 300 V nozzle voltage.

The separation column was a Kromasil 60-5-HILIC-D column (2.1 × 150 mm; 5 μm particle size; Nouryon, Bohus, Sweden), equipped with a Kromasil 60-5-HILIC-D guard column (2.1 × 10 mm; 5 μm particle size; Nouryon, Bohus, Sweden). The column was maintained at 30 °C and a flow of 0.3 mL min^−1^ was employed. Injection volume was 2 μL.

A ternary gradient was employed with (A) 5 mM ammonium formate in H_2_O containing 0.1% formic acid; (B) acetonitrile with 0.1% formic acid and (C) 100 mM ammonium formate in H_2_O containing 0.1% formic acid. The following gradient conditions were applied: starting with 4% A, 96% B, held constant for 2 min; from 2 to 8 min linear increase to 43% A, 55% B, 2% C, constantly held for 4 min. Within 0.5 min, the gradient was changed to the starting conditions and held for 7.5 min for re-equilibration of the column.

### Preparation of model samples

For method development and calibration, model samples were prepared by compounding the polyolefin (high- or low-density polyethylene, polypropylene) with PA 6. The PA 6 concentrations used were 0.2, 0.5, 1, 2.5 and 5 wt% respectively.

### Sample extraction

One hundred milligrams sample was weighed in G4 reaction vessels (Anton Paar GmbH, Graz, Austria) and 2 mL solvent (methanol/toluene, 50/50 (v/v)) was added. The microwave-assisted extraction was performed with a Monowave Extra equipped with a MAS24 autosampler (Anton Paar GmbH, Graz, Austria). The temperature was held at 160 °C for 20 min and constant stirring at 600 rpm was applied. Subsequently, the samples were filtrated through a Chromafil AO-45/25 RC filter (Macherey Nagel, Düren, Germany). Five hundred microliters of each sample was evaporated to dryness under a nitrogen stream and reconstituted with 500 μL acetonitrile. All samples were prepared in triplicates and stored in a refrigerator at 6 °C until the analysis by HPLC.

### Data evaluation

Data acquisition was done using Agilent MassHunter LCMS Acquisition software 10.0. For the optimization of the QQQ-MS parameters, MassHunter Optimizer 10.0 and MassHunter Source Optimizer 10.0 were used. The software Agilent MassHunter Qualitative Analysis B.07.00 and Agilent MassHunter Quantitative Analysis 10.1 were employed for data evaluation.

## Results and discussion

### Development of the extraction procedure

Microwave-assisted extraction was chosen as being a rapid method compared to an extraction at room temperature. The extraction solvent needed to fulfil two requirements, a high dielectric constant for the absorption of the microwaves and a sufficient swelling of the polyolefin to guarantee the migration of low-molecular weight compounds of PA 6 into the solvent. As no single solvent fulfilled both requirements, solvent mixtures of polar and apolar solvents were tested leading to the result that a mixture of toluene and methanol was best suited. Solvent ratios, extraction time and extraction temperature were optimized. Toluene leads to a swelling of the polyolefin, and the addition of the polar methanol allows a satisfactory extraction of the analytes. The final extraction was performed by the usage of toluene/methanol in a ratio of 50/50 (v/v) at 160 °C for 20 min. Thereby, a pressure of 18 bar was reached in the reaction vessel.

### Identification of marker compounds

**Fig. 1:**
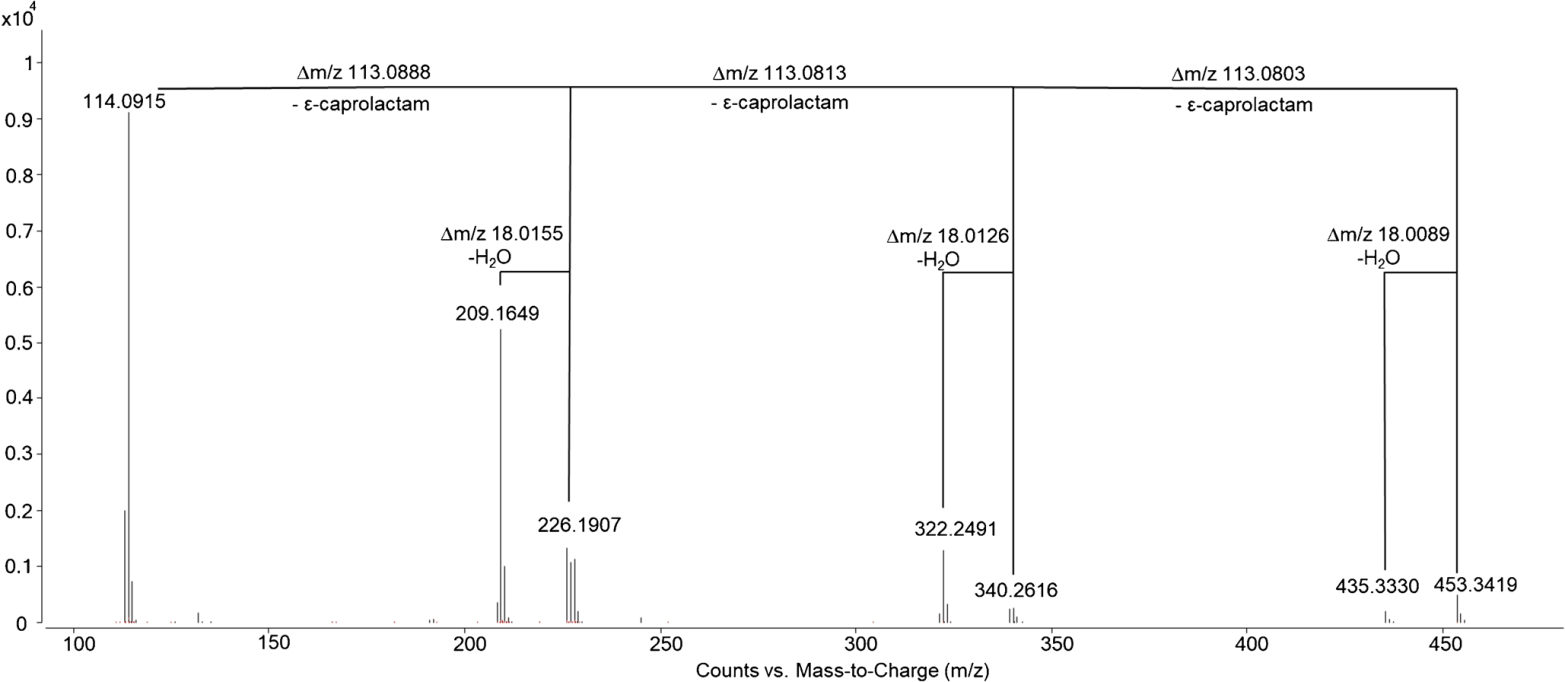
MS/MS spectrum of the cyclic tetramer, recorded with a collision energy of 40 V

Method development was done using model samples prepared by compounding a polyolefin with various amounts of PA 6. Results obtained by preliminary experiments using HPLC (with a not yet fully optimized mobile phase) and QTOF-MS showed the presence of different cyclic compounds (Table 1) which can be related to PA 6, namely ε-caprolactam and the cyclic di- to hexamer. For the verification of the compounds targeted MS/MS experiments were performed with the QTOF-MS, due to unavailability of standards. Collision energies ranging from 0 to 60 V were applied for fragmentation; fragmentor voltage was kept constant at 125 V. Thereby, a fragmentation pattern could be observed for the investigated cyclic compounds. ε-Caprolactam as the smallest fragment as well as the corresponding lower cyclic oligomers with respect to the chosen precursor ion was found. These fragments could be detected without or with a water loss. As an example, the MS/MS spectrum of the cyclic tetramer (collision energy 40 V) can be seen in Fig. 1. The spectra of the other cyclic compounds can be found in the Electronic Supplementary Material.

**Table 1:**
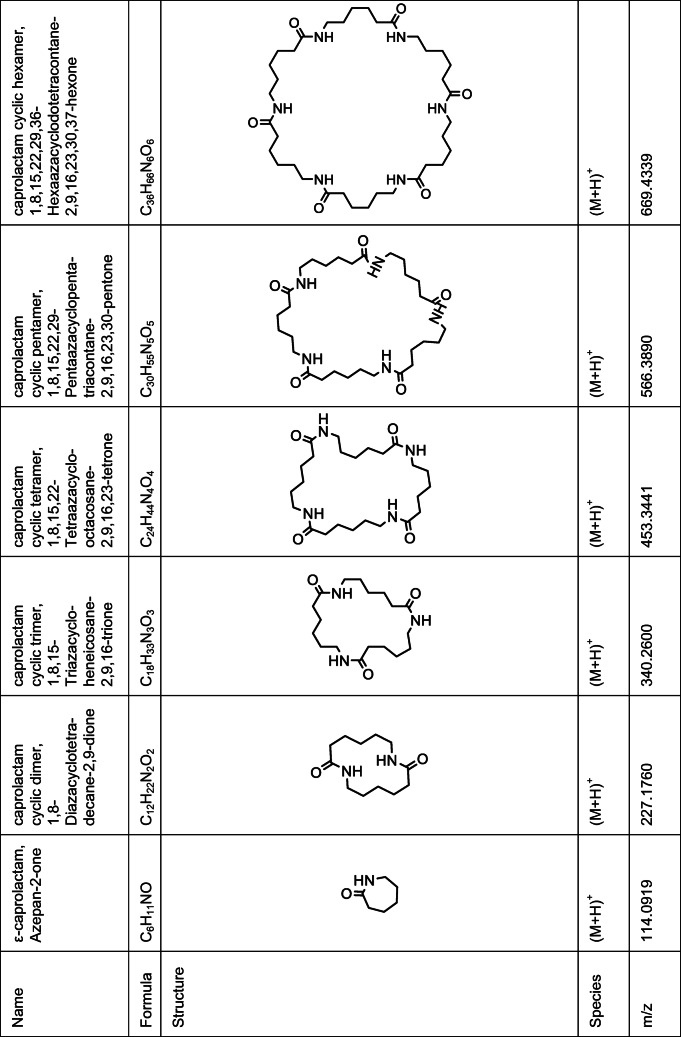
Description of the compounds identified by QTOF-MS

### Optimization of HPLC conditions

A HILIC column was chosen for the separation of the marker compounds, due to the fact that RP columns show co-elution and separation problems, such as the elution of ε-caprolactam peak in the void volume, an insufficient separation of the cyclic pentamer and hexamer as well as poor peak shapes due to peak tailing. Several attempts for optimization only yielded unsatisfying results.

**Fig. 2:**
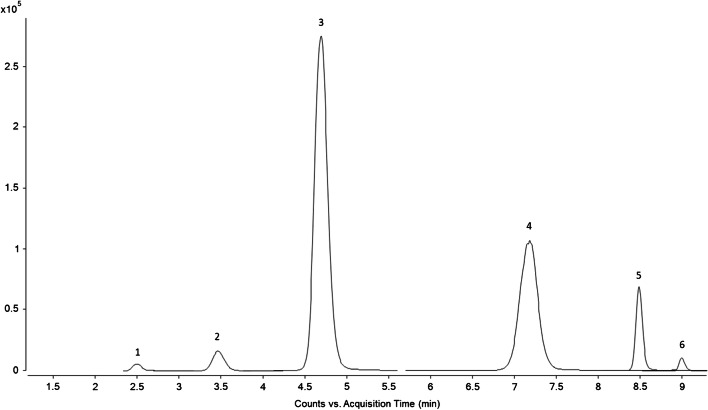
Extracted ion chromatograms of the ε-caprolactam monomer to the hexamer of a HDPE sample, containing 1 wt% PA 6. Peaks: (1) ε-caprolactam, (2) cyclic dimer, (3) cyclic trimer, (4) cyclic tetramer, (5) cyclic pentamer, (6) cyclic hexamer

Separation with a HILIC column offered several advantages like good peak shape and sufficient separation of the peaks, and the non-polar polyolefin matrix does not affect the chromatography. The gradient for the separation was optimized, firstly because of little retention of ε-caprolactam. Several experiments showed a relation between the salt concentration in the mobile phase at the start of the gradient and the retention of the monomer, whereby higher concentrations lead to lower retention times. Therefore, starting conditions without eluent C were selected. Another optimization was needed for the separation of the cyclic pentamer and hexamer. Thereby, eluent C with a higher salt concentration allowed a better separation of the cyclic pentamer and hexamer and improved the peak shape as well. Optimum conditions were achieved with 2% of eluent C and higher concentrations lead to lower retention times and again co-elution. Figure 2 shows the optimized separation of the analytes in a HDPE sample contaminated by 1 wt% PA 6.

### Quantitation

The quantitative analysis was performed on the QQQ-MS, starting with the optimization of the fragmentor voltage and collision energies for each analyte, using the MassHunter Optimizer software. Fragmentor voltage was varied from 30 to 300 V, collision energies from 4 to 40 V. Cell accelerator voltage was kept constant at 7 V. For each analyte, two qualifier ions were chosen as verification, except for the cyclic hexamer, due to low abundances. The resulting parameters are displayed in Table 2.

**Table 2:** MS parameters for the MRM transitions

Compound	Formula	Molecular weight (g mol^−1^)	Retention time (min)	Precursor ion	Product ion	Fragmentor voltage (V)	Collision energy (V)
ε-Caprolactam	C_6_H_11_NO	113.16	2.5	114.1	44.0*	290	24
					79.0	290	69
					69.1	290	12
Cyclic dimer	C_12_H_22_N_2_O_2_	226.32	3.5	227.2	96.1*	250	20
					69.0	250	28
					41.0	250	40
Cyclic trimer	C_18_H_33_N_3_O_3_	339.48	4.6	340.3	322.2*	138	12
					96.1	138	32
					209.1	138	24
Cyclic tetramer	C_24_H_44_N_4_O_4_	452.64	7.2	453.4	435.4*	84	20
					209.2	84	36
					322.2	84	28
Cyclic pentamer	C_30_H_55_N_5_O_5_	565.80	8.6	566.4	548.4*	198	24
					209.2	198	48
					322.2	198	36
Cyclic hexamer	C_36_H_66_N_6_O_6_	678.96	9.1	679.5	661.3*	254	28
					209.2	254	48

*Used as quantifiers

For calibration of the method, model samples described in the “Materials and methods” section were used. The compounded samples were measured in triplicates. For each polymer, an individual calibration was performed using linear regression. The resulting slopes, intercepts, measures of certainty, standard deviations of the method, and limits of detection (LODs) and limits of quantification (LOQs) were calculated (Table 3). The highest abundances were found for the cyclic trimer, followed by the cyclic tetramer, leading to lower LODs and LOQs for PA contaminations compared to the other analytes. Therefore, these signals are best suited for the quantification of PA 6 in polyolefins. The others can be used as qualifiers for the presence of PA 6 in order to avoid false positives. The cyclic hexamer was excluded due to low abundances and irreproducible results. By comparison of the LOQs of the different polyolefins, it can be noticed that ε-caprolactam and the cyclic pentamer show higher LOQs than the cyclic dimer to tetramer. The explanation for the monomer is its lower abundance of one order in magnitude compared to the cyclic trimer. The cyclic pentamer shows an increased method standard deviation, which has a significant impact on the calculation of the LOD and LOQ.

**Table 3:** Results of the quantitative analysis of the compounded samples, measured with the QQQ-MS

Compound	Slope (sensitivity)	Intercept	Method standard deviation	*R*^2^	LOD	LOQ
	Peak area wt%^−1^	Peak area	wt%		wt%	wt%
HDPE						
ε-Caprolactam	6.150·10^3^	3.735·10^3^	0.10	0.9878	0.19	0.53
Cyclic dimer	6.901·10^4^	5.930·10^3^	0.04	0.9982	0.07	0.21
Cyclic trimer	4.121·10^6^	4.340·10^5^	0.01	0.9997	0.03	0.08
Cyclic tetramer	3.418·10^6^	− 2.401·10^4^	0.02	0.9996	0.03	0.10
Cyclic pentamer	9.076·10^5^	4.106·10^5^	0.21	0.9522	0.37	1.00
LDPE						
ε-Caprolactam	3.783·10^3^	2.150·10^3^	0.34	0.8838	0.61	1.57
Cyclic dimer	7.875·10^4^	− 1.098·10^4^	0.11	0.9865	0.20	0.55
Cyclic trimer	4.049·10^6^	− 4.267·10^4^	0.08	0.9925	0.15	0.42
Cyclic tetramer	3.291·10^6^	− 5.652·10^5^	0.13	0.9817	0.23	0.64
Cyclic pentamer	7.039·10^5^	3.181·10^5^	0.22	0.9476	0.39	1.04
PP						
ε-Caprolactam	1.428·10^4^	5.867·10^3^	0.14	0.9789	0.25	0.68
Cyclic dimer	1.016·10^5^	− 1.011·10^4^	0.06	0.9956	0.11	0.32
Cyclic trimer	4.989·10^6^	− 3.340·10^5^	0.03	0.9992	0.05	0.14
Cyclic tetramer	4.601·10^6^	− 7.046·10^5^	0.04	0.9987	0.06	0.21
Cyclic pentamer	1.023·10^6^	4.784·10^5^	0.36	0.8726	0.64	1.65

Alternatively, one might consider the use of caprolactam for quantitation of the oligomers, taking into account the number of monomers in each oligomer. Such an approach requires that the response factors of caprolactam and of the building block in the oligomers are the same. Unfortunately, this is not the case at all. A QQQ-MS was employed, and thereby, the responses of specific transitions to specific fragments are used. These responses are completely different to the response of caprolactam.

A quality control sample was measured several times within every sequence of sample injections to observe whether the sensitivity of the QQQ-MS changes over time. Overall, a standard deviation of 5% could be detected, without any trend of drifting results, leading to the assumption that the sensitivity of the instrument is constant over time.

To reduce the probability of false positives, method blanks, using the pure polyolefins (*n* = 3) or only the solvents (*n* = 10), were measured. There can be signals detected for the cyclic tri- to hexamer, yet all of them far below the LOD. The source of contamination was searched for by analysing every step of sample preparation and all used solvents but without satisfying results. A carry-over by the instrument was excluded as well, due to the fact that there are no detectable signals in ACN blanks, measured after every tenth sample injection.

As a prerequisite for the reliability of the method, PA 6 must not degrade under the conditions of the microwave-assisted extraction. To prove the absence of such artefacts, the 5 wt% PA 6 sample of each polyolefin type was kept in the extraction solvent at room temperature for an extended period of time. The results demonstrated that the pattern of oligomers in the samples kept at room temperature is the same as in case of microwave-assisted extraction. After 12 days of treatment at room temperature, the extracted amounts of the cyclic tri- to pentamer approached those obtained with microwave-assisted extraction. These results confirm that the cyclic oligomers are indeed present in the recyclate samples and are not formed at the elevated temperature of the microwave-assisted extraction. Yet, the amount of the cyclic dimer was significantly higher in the extract kept at room temperature, compared to the microwave-assisted extraction and inversely for the monomer. To prove if these results are related to the energy impact of the microwave, the extracted solution at room temperature was microwaved afterwards. The results for the cyclic tri- to pentamer were constant but the cyclic dimer was partly decomposed during the extraction and formed ε-caprolactam.

Another question must be critically taken into account, namely if PA contaminants present in different polyolefin recyclates would contain the same percentage of cyclic oligomers. Therefore, another two PA 6 materials from different vendors were compared to the one which was used for the preparation of the polyolefin-PA 6 model samples. The results showed that the relative standard deviation is varying for the different marker compounds. The variation of the analytes with higher molecular weight is lower compared to the ε-caprolactam or the cyclic dimer. The monomer content in PA 6 is dependent on the polymerization process parameters as well as on the applied post-treatment for the monomer removal. Mainly hot-water extraction is used, yet the residual concentration can differ due to extraction time or temperature. Cyclic oligomers are hardly affected by this process [21]. Therefore, 40% relative standard deviation for ε-caprolactam can be easily explained. Comparing the results for the cyclic trimer and the cyclic tetramer used for the quantitation, the relative standard deviations calculated were 20% for the trimer and 14% for the tetramer, respectively. Even lower standard deviations were found for the cyclic pentamer and hexamer, 11% and 4%. This leads to the assumption that the formation of cyclic compounds with higher molecular weight is less affected by the polymerization process and that the concentrations do not decrease significantly by the post-extraction treatment.

To guarantee that the distribution of the signal intensities of the marker compounds is not affected by the temperature and mechanical stress applied in the recycling process, a 1 wt% PA 6 in HDPE sample was extruded several times at a temperature of 220 °C, using a speed of 100 rounds per minute. Samples were drawn at minutes 5, 10, 15, 20 and 25. The results showed that there exists no significant change over several extrusion cycles as the observed variations were within the standard deviation of the method. No trends towards higher signal intensities due to degradation of the PA 6 could be observed. Therefore, it can be noted that the method is reliable and independent of any applied stress during the recycling process.

To prove the applicability of the method, six samples from various hardware stores were measured in triplicates. Cyclic polyamide compounds could be detected in three out of six samples. All of these samples consisted of recycled HDPE. After quantification using the calibration of the cyclic trimer and the cyclic tetramer, amounts between 0.7 and 1.3 wt% PA 6 in the samples could be observed.

## Conclusion

The aim of the present work was the development of a HPLC-MS-method for the quantitative determination of PA 6 contaminations in polyolefin recyclates. Therefore, a microwave-assisted extraction was employed, cyclic oligomers were identified as marker compounds for PA 6 and a complete separation of these cyclic oligomers were achieved by the employment of a HILIC column, using a ternary gradient. For the quantitation, model compounds with defined proportion of PA 6 were used as standards for external calibration. Via further experiments, it was confirmed that these marker compounds are already present in the PA 6 and not formed during the extraction process. The developed method is applicable down to 0.2 wt% PA 6 contamination in polyolefins. Via simulation of the recycling process and measurements of real samples, it could be shown that the developed method is applicable for routine quality control of recycled polyolefin materials.

## Supplementary Information

ESM 1 (PDF 420 kb)
