# Supplementary material for: Development of an LC-MS method for the semiquantitative determination of polyamide 6 contaminations in polyolefin recyclates
Source: Anal Bioanal Chem. 2020 Nov 26;413(4):1091–8. doi: 10.1007/s00216-020-03071-z (PMC7813719; doi:10.1007/s00216-020-03071-z)

Analytical and Bioanalytical Chemistry

Electronic Supplementary Material

Development of an LC-MS method for the semiquantitative determination of polyamide 6 contaminations in polyolefin recyclates

Andrea Schweighuber,*[1] Markus Gall,[2] Jörg Fischer,[2] Yi Liu,[3] Hermann Braun,[3] Wolfgang Buchberger[1]

MS/MS data of the cyclic dimer

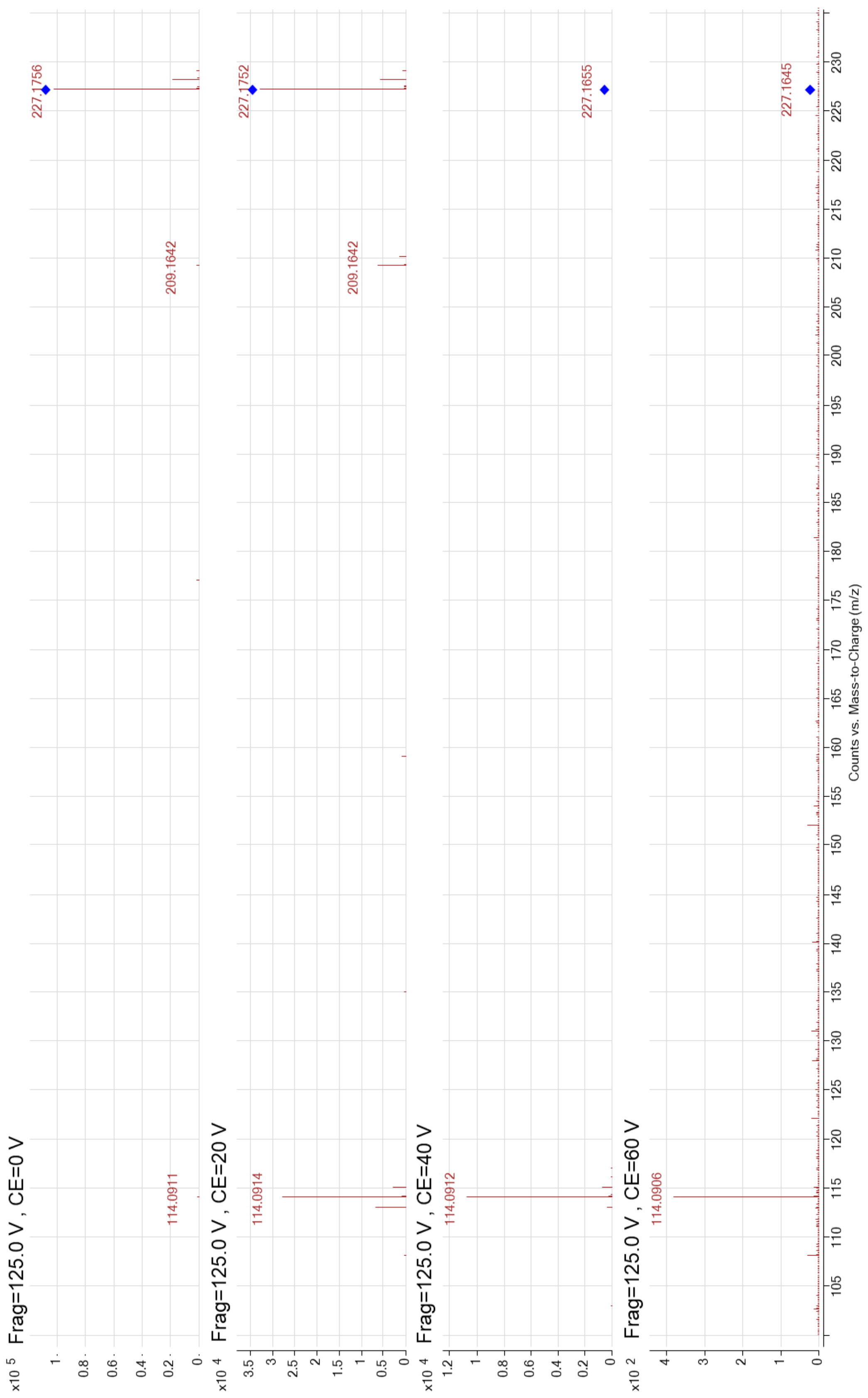

MS/MS data of the cyclic trimer

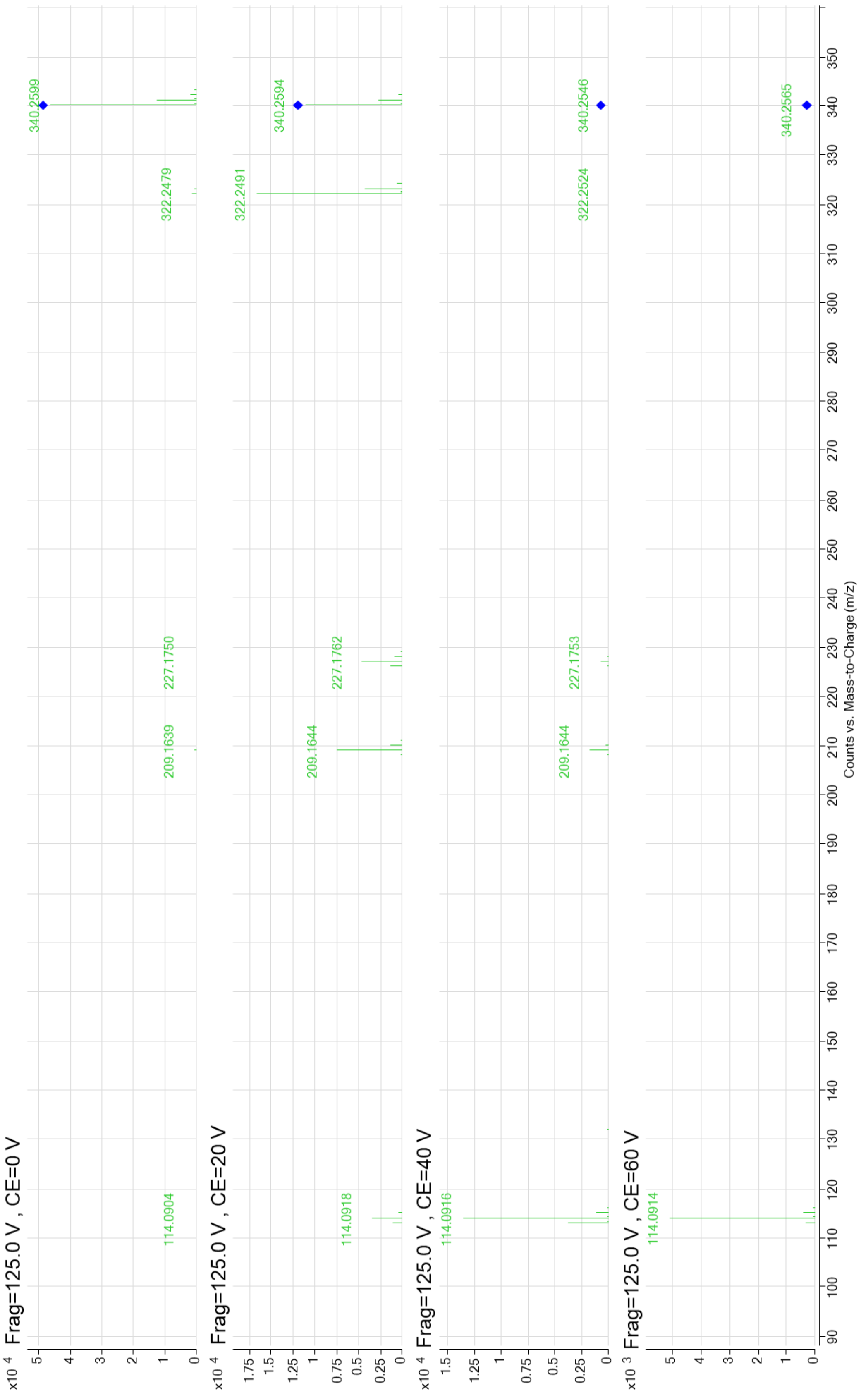

MS/MS data of the cyclic tetramer

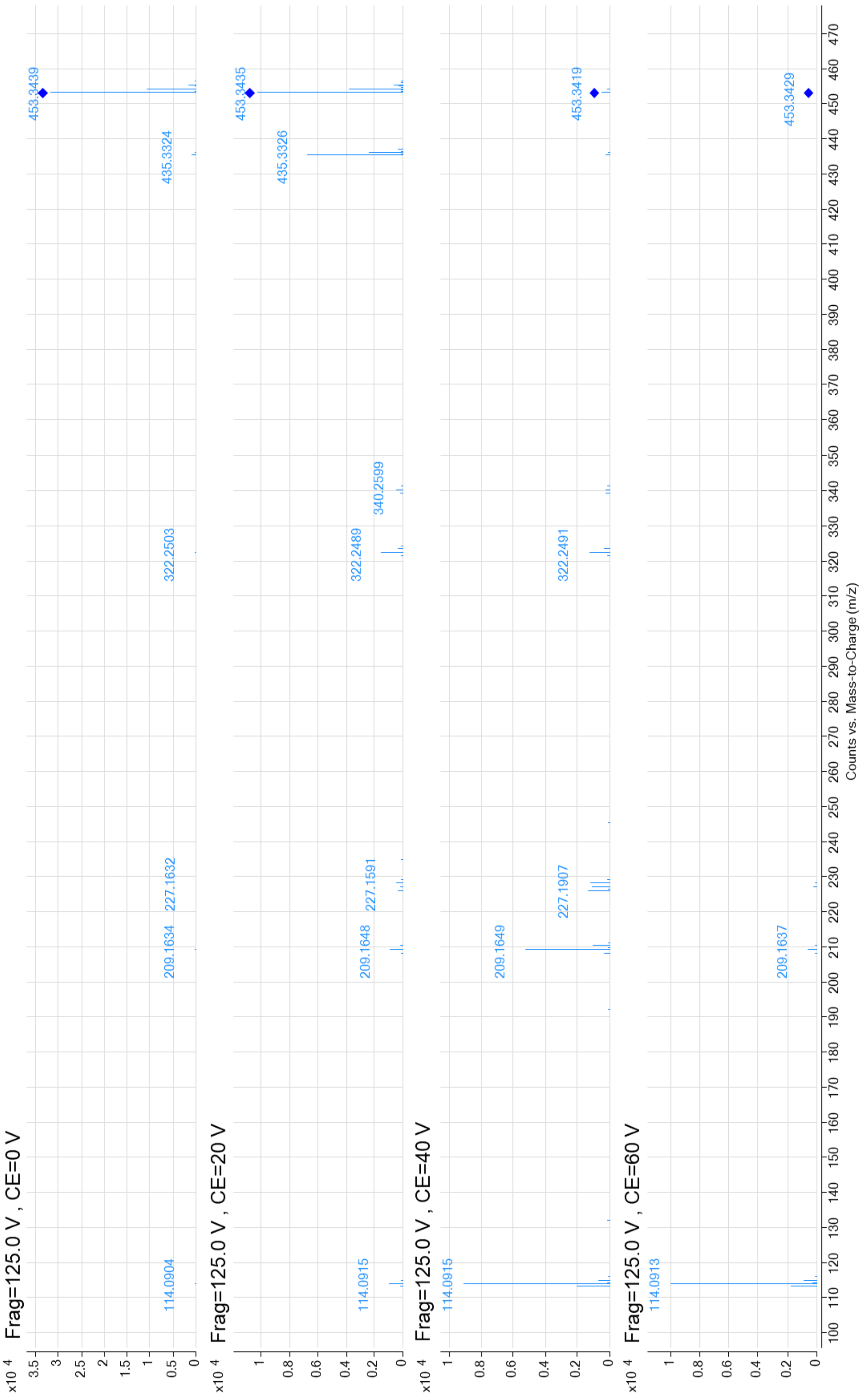

MS/MS data of the cyclic pentamer

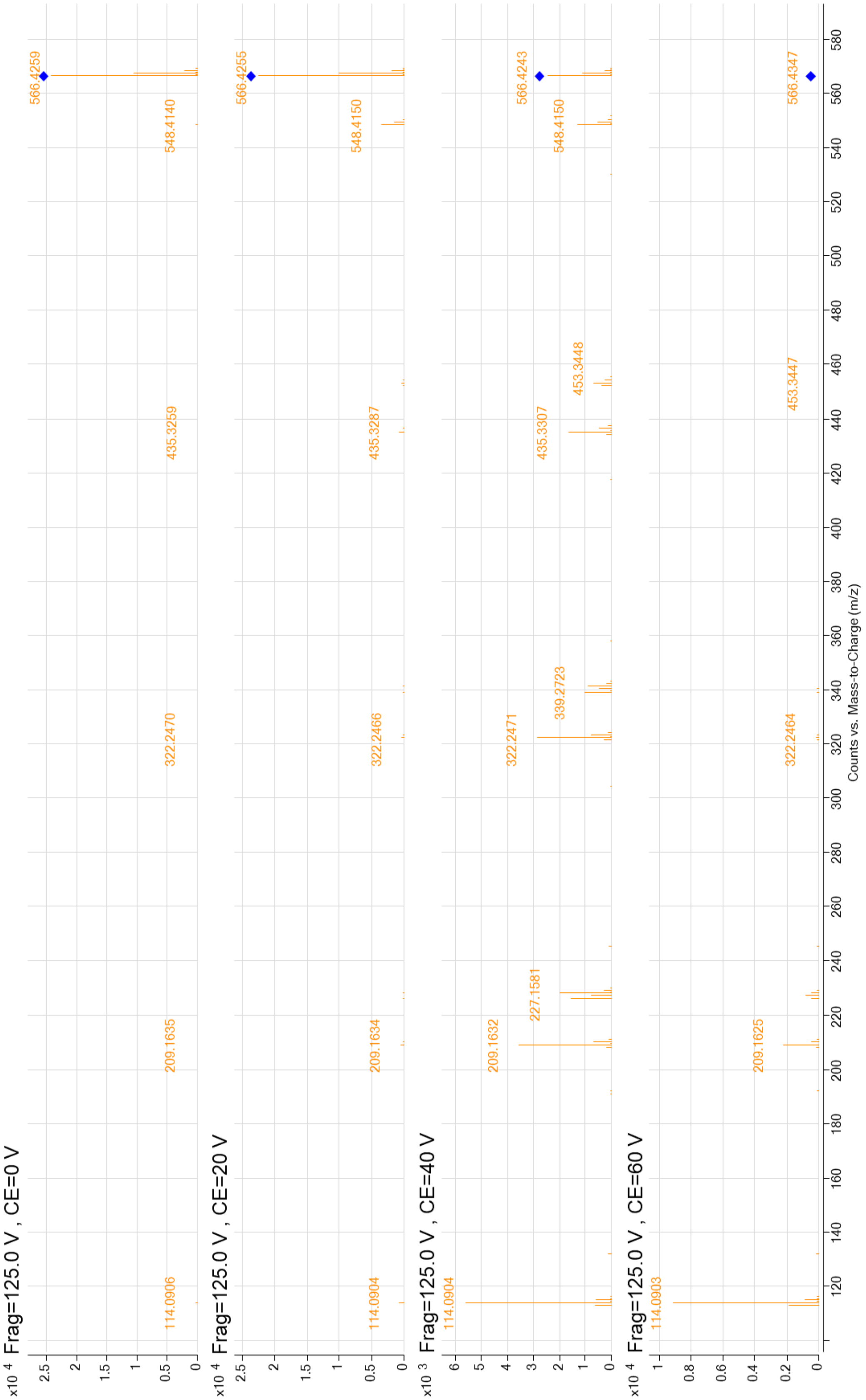

MS/MS data of the cyclic hexamer

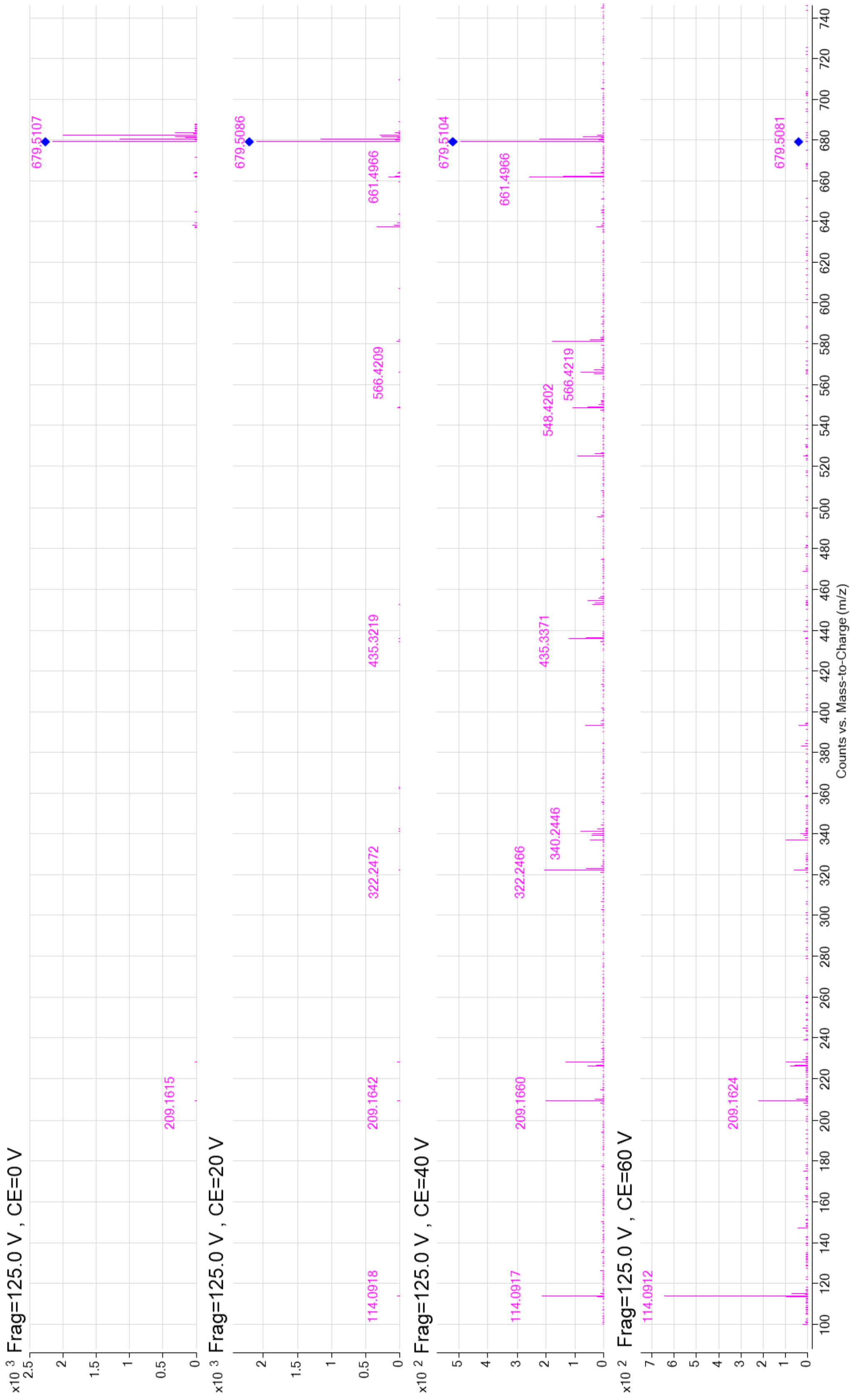

Supplement: Supplementary file 1 — (PDF 420 kb) [file 216_2020_3071_MOESM1_ESM.pdf]
